# Radical radiotherapy in patients with cervix uteri carcinoma: experience of Ondokuz Mayis University

**DOI:** 10.1186/s12885-019-6402-x

**Published:** 2019-12-12

**Authors:** Alparslan Serarslan, Bilge Gursel, Deniz Meydan, Nilgun Ozbek Okumus

**Affiliations:** Department of Radiation Oncology, Faculty of Medicine, Ondokuz Mayis University, Kurupelit, 55139 Atakum/Samsun, Turkey

**Keywords:** Brachytherapy, Cervix cancer, Chemotherapy, Radiotherapy

## Abstract

**Background:**

Radical radiotherapy is the standard treatment for patients with locally advanced cervix uteri carcinoma (FIGO stage IB2–IVA). Worldwide, incidence and mortality rates vary among regions because of differences in lifestyles and treatment standards. Herein, we evaluated the outcomes of radical radiotherapy in patients with locally advanced cervix uteri carcinoma from the middle Black Sea region of Turkey.

**Methods:**

We retrospectively reviewed the records of 64 consecutive patients with locally advanced cervix uteri carcinoma who were treated from January 2013 to 2016 in our radiation oncology department. All patients staging and radiotherapy planning were performed with modern imaging techniques including magnetic resonance imaging and positron-emission-tomography/computed tomography before radical radiotherapy. Thereafter, all of them were treated with external beam radiotherapy and concurrent cis-platinum-based chemotherapy followed by three-dimensional intra-cavitary high-dose-rate brachytherapy.

**Results:**

The median age at diagnosis was 54.5 years. The median follow-up period was 21 months. Acute grade 3 toxicity was detected in 3.1% of patients. Late toxicity was not detected in any patient. The 1- and 3-year progression-free survival rates were 83.6 and 67.5%, respectively. The 1- and 3-year overall survival rates were 95.7 and 76.9%, respectively. The most important prognostic factor was the FIGO stage. Distant metastasis was the most common cause of death in patients with locally advanced cervix uteri carcinoma despite radical radiotherapy.

**Conclusions:**

In patients with locally-advanced cervix uteri carcinoma from the middle Black Sea region of our developing country, acceptable toxicity and survival rates are achieved similar to the recent literature from developed countries with using of modern staging, planning and radical radiotherapy techniques. However, recurrence was mostly in the form of distant metastases and further investigations on systemic therapies are required.

## Background

Cervix uteri carcinoma is the most common gynecological cancer worldwide. In addition, it is the fourth most common malignancy and the fourth leading cause of cancer-related death in women. Incidence and death rates of cervix uteri carcinoma shows significant geographical variation [1, 2]. In Turkey, cervix uteri carcinoma is the third most common gynecological cancer, after corpus uteri and ovarian carcinoma. In addition, it is the ninth most common malignancy in women [3]. Although the incidence is slightly lower than that in western countries (4.2 vs. 7.1 per 100,000), the mortality rate is slightly higher (1.8 vs. 1.6 per 100,000) [4]. This variation may be due to religious, social, cultural, and economic differences.

Cervix uteri carcinoma is diagnosed mostly (> 80%) at the locally advanced stages [Fédération Internationale de Gynécologie et d’Obstétrique (FIGO) stage IB2-IVA and/or node-positive] in developing countries such as Turkey [3, 5]. External beam radiotherapy (EBRT) with concurrent platinum-based chemotherapy (CHT) followed by brachytherapy (BRA), also known as radical radiotherapy (RT), is the standard treatment for locally advanced cervix uteri carcinoma (LACC), based on the results of five-randomized-controlled trials [6–11].

No studies have explored the treatment outcomes of cervix uteri carcinoma in Turkey. The Radiation Oncology Department of Ondokuz Mayis University Hospital is the only center with a BRA device and treats LACC patients from the Black Sea region of Turkey. Therefore, we retrospectively analyzed radical RT outcomes in patients with LACC from the Black Sea region of Turkey.

## Methods

### Ethics, consent and permissions

This study was approved by the local Ethics Committee of Ondokuz Mayis University Hospital, Samsun, Turkey (acceptance date: 22/6/2017; and number: 2017/256), and all patients provided written inform consent.

### Patient evaluation

A total of 64 patients with FIGO stage IB2-IVA and/or node metastasis positive (N+) cervix uteri carcinoma, treated with radical RT from January 2013 to December 2016, were included in this retrospective study. All patients were initially evaluated using abdominopelvic magnetic resonance imaging (MRI) and whole-body positron emission tomography-computed tomography (PET-CT). Patients with paraaortic lymph node metastasis were excluded from the study. All patients had normal hematological (white-blood-cell count, 3000–10,000/μl; hemoglobin, > 10 g/dl; platelet count, ≥ 100,000/μl), renal (creatinine clearance [CrCl], ≥ 60 ml/min), and hepatic (bilirubin level, ≤ 1.5 mg/dl; aspartate aminotransferase and alanine aminotransferase levels, ≤ 2.5 × the upper limit of normal) function.

### Simulation

EBRT and high dose rate (HDR)-BRA planning was performed three-dimensionally using a computed tomography (CT) simulator (Asteion Super 4; Toshiba Medical Systems, Otawara, Japan). Patients were immobilized in the supine position. CT imaging was under free breathing, full bladder, and empty rectum conditions at a slice thickness of 3 mm. For the HDR-BRA procedure, a tandem ring (TR) applicator was used in conjunction with a rectal retractor. Additionally, a Foley’s catheter was placed in the bladder and its balloon was filled with 7 ml of diluted contrast media. Normal saline solution (90 ml) plus 10 ml of contrast medium was added to the bladder to determine the bladder volume for treatment planning. The datasets were transferred to a treatment planning system (TPS; Eclipse 8.6 for EBRT and 10.0 for HDR-BRA; Varian Medical Systems, Palo Alto, CA, USA) via a digital imaging and communications in medicine (DICOM) network.

### Target volumes and delineation of organs at risk

The gross tumor volume (GTV), clinical target volume (CTV), planning target volume (PTV), and organs at risk (OAR) were defined using individual axial CT slices. For the EBRT planning, CTV-1 included the GTV, corpus uteri, cervix uteri, common iliac, external iliac, internal iliac, obturator, and presacral lymph nodes, and at least 3 cm of the vaginal margin from the GTV. CTV-2 included the parametrium. PTV-1 and PTV-2 were defined as additional 1.5 cm and 1 cm margins around CTV-1 and CTV-2, respectively. PTV-total consisted of PTV-1 and PTV-2. For the HDR-BRA planning, Groupe Européen de Curiethérapie and the European Society for Radiotherapy and Oncology (GEC-ESTRO) recommendations were used to define the target volumes (e.g., high-risk CTV = HR-CTV). HR-CTV was delineated using individual axial CT slices based on post-EBRT MRI, and 3D conformational planning was used for BRA.

### External beam radiotherapy and concurrent chemotherapy

The prescribed dose of EBRT to the PTV-total was 45–50.4 Gy in 25–28 fractions for 5 to 5.5 weeks, using the three-dimensional (3-D) conformal RT (3D-CRT) technique (four-field) with 18 MV photon energy with a linear accelerator (Clinac DHX; Varian Medical Systems, Palo Alto, CA, USA). The prescribed dose selection (45 or 50.4) was administered at the discretion of the treating physician. The aim of the target coverage was to deliver at least 95 and 100% of the prescribed dose to the PTV and CTV, respectively. The mean dose was constrained to < 50 Gy for the rectum, with a maximum dose of 52 Gy for the small intestine, and of 60 Gy for the bladder. Concurrent CHT comprised 40 mg/m^2^/day cisplatin (DDP) per week. The CHT was omitted if the white blood cell and neutrophil counts were below 3000 cells/μl and 1500/μl, respectively, or the platelet count was below 100,000/μl. Complete blood count, kidney, and liver functions were assessed twice a week, including once before CHT. The DDP dose was modified according to the weekly CrCl value. The DDP dose was reduced when CrCl was < 40 ml/min and administration was stopped when it was < 30 ml/min.

### Brachytherapy

After finishing EBRT, a dose of 5.5 Gy per fraction, in five fractions, was delivered to the HR-CTV by intracavitary HDR-BRA with an iridium-192 source using a BRA afterloader (GammaMedplus IX HDR Afterloader; Varian Medical Systems, Palo Alto, CA, USA), once a week under conscious sedation for all patients. The dose was constrained to D_2cc_ < 75 Gy for the rectum, < 75 Gy for the sigmoid colon, and < 90 Gy for the bladder.

### Follow-up

After completing radical RT, initial tumor control was completed in all patients after 6 weeks of abdominopelvic MRI and 12 weeks of PET-CT. Thereafter, patients were followed-up at 3-month intervals for the first 2 years, biannually for years 2–5, and annually thereafter. Abdominopelvic MRI and cervical cytology were performed every 6 months for the first 5 years and annually thereafter. Early and late toxicity grading were performed according to toxicity criteria of the Radiation Therapy Oncology Group (RTOG) and the European Organization for Research and Treatment of Cancer (EORTC).

### End points and statistical analysis

Local control, regional control, death from any cause or from disease, locoregional failure-free survival (LRFFS), distant metastasis-free survival (DMFS), progression-free survival (PFS), and overall survival (OS) were recorded and calculated. LRFFS was defined as the time between the date of diagnosis and the date of first local or regional relapse. DMFS was defined as the time between the date of diagnosis and the date of first distant metastasis. PFS was defined as the time between the date of diagnosis and the date of the first failure at any site. OS was defined as the time between the date of diagnosis and the date of death from any cause. FIGO stage, node metastasis, parametrial invasion, tumor size, initial tumor volume, size of HR-CTV, number of concurrent CHT course and D90 (EQD2) were potential factors affecting survival. Patient characteristics were described using descriptive statistics. The Kaplan-Meier method was used to analyze survival and the log-rank test was used for univariate analysis. Multivariate Cox regression analysis was performed on the significant determinants identified by univariate analysis. A value of *P* < 0.05 was considered statistically significant.

## Results

### Patient clinical characteristics

All 64 patients were from the Black Sea region of Turkey. The median age at diagnosis was 54.5 ± 13.6 years (range: 28–81 years). The median weight was 71 ± 13.7 kg (range: 48–94 kg). The histopathological diagnosis was squamous-cell carcinoma in 58 (90.6%) patients. The FIGO stage was 2B or higher in more than three-quarters (*n* = 51; 79.5%) of the patients. Lymph node metastasis was present in 35 (54.6%) patients (Table 1). The median maximum diameter of the tumor was 5.1 ± 1.4 cm (range: 2.5–10 cm). The median tumor volume was 64.5 ± 56.1 cm^3^ (range: 6.3–310 cm^3^). Parametrial invasion was present in 50 (78.1%) patients.

**Table 1:** Clinical characteristics of the patients (*n* = 64)

	Patients’ number	%
Histopathology		
Squamous cell carcinoma	58	90.6
Adenocarcinoma	3	4.7
Adeno-squamous cell carcinoma	1	1.6
Clear cell carcinoma	1	1.6
Small cell carcinoma	1	1.6
FIGO Stage		
IB2	6	9.4
IIA	7	10.9
IIB	29	45.1
III	20	31.3
IV	2	3.1
Node metastasis		
Negative	29	45.4
Positive	35	54.6

*FIGO* Fédération Internationale de Gynécologie et d’Obstétrique

### Treatment

The median time from diagnosis to EBRT was 32.5 ± 21.6 days (range: 7–96 days). The EBRT dose was 45 Gy in 48 (75%) patients and 50.4 in 16 (25%) patients. All patients completed the planned EBRT. Of the 64 patients treated with weekly DDP, 45 (70.3%) received five or more, and 13 (20.3%) received between one and five, cycles. However, in six (9.4%) patients, DDP was not administered due to high levels of blood creatinine or low CrCl. BRA was applied to all patients.

### Dose-volume parameters

The mean HR-CTV at the time of the first BRA was 31.56 ± 13.15 cm^3^. The mean HR-CTV dose was 90.16 ± 8.15 equivalent dose in 2 Gy (EQD2). The mean D_2cc_ dose for the rectum, sigmoid, and bladder was 65.51 ± 7.02, 63.37 ± 8.79, and 82.40 ± 5.84 EQD2, respectively.

### Patterns of failure

There were 12 (18.7%) relapses in total; 4 (6.2%) patients relapsed loco-regionally (local failure, *n* = 2; regional failure, *n* = 1; loco-regional failure, n = 1), 7 (11%) showed distant metastases, and 1 (1.5%) showed both loco-regional and distant metastasis. Two-thirds of metastases were in the lungs. Ten (15.6%) patients had died by the end of the follow-up period. All deaths were due to cervix uteri carcinoma. The cause of death was local-regional failure in two (3%) patients and distant metastasis in the remaining eight (12.6%) patients.

### Toxicity

Acute gastrointestinal system (GIS) toxicity was grade 0 in 48 (75%), grade 1 in 6 (9.4%), and grade 2 in 10 (15.6%) patients. Acute hematologic system toxicity was grade 0 in 44 (68.8%), grade 1 in 4 (6.3%), grade 2 in 14 (21.9%), and grade 3 in 2 (3.1%) patients. Acute genitourinary system (GUS) toxicity was not detected. Acute (grade 4 or higher) and late toxicity were not detected in any patients.

### Survival

After a median follow-up period of 21 months (range: 3–60 months), 54 (84%) patients were alive. The 1- and 3-year LRFFS rates were 96 and 87%, respectively. The 1- and 3-year DMFS rates were 94.4 and 76%, respectively. The 1- and 3-year PFS rates were 83.6 and 67.5%, respectively. The 1- and 3-year OS rates were 95.7 and 76.9%, respectively.

### Prognostic factors

In the univariate analysis, significant prognostic factors for LRFFS included FIGO stage (*P* = 0.003) and D90 (EQD2) dose (*P* = 0.01). The 3-year LRFFS rate according to FIGO stage ≤IIA, IIB, III, and IV was 100, 95, 71.3, and 50%, respectively. The 3-year LRFFS rate according to a D90 (EQD2) dose ≤90 Gy and > 90 Gy was 65 and 100%, respectively. In addition, significant prognostic factors for PFS and OS included FIGO stage (*P* = 0.01 and *P* = 0.03, respectively) and lymph node status (*P* = 0.008 and *P* = 0.001, respectively). The 3-year PFS rate according to FIGO stage ≤ IIA, IIB, III, and IV was 100, 75.9, 46, and 50%, respectively. The 3-year PFS rate according to lymph node status, i.e., node-negative versus pelvic node-positive, was 79.2 and 73.1%, respectively. The 3-year OS rate according to FIGO stage ≤ IIA, IIB, and III was 100, 88.1, and 52.6%, respectively. The 3-year OS rate according to lymph node status, i.e., node-negative versus pelvic node-positive, was 91.7 and 74.6%, respectively (Table 2). In the multivariate analysis, the only significant prognostic factor for LRFFS, PFS, and OS was the FIGO stage (*P* = 0.04, *P* = 0.01, and *P* = 0.02, respectively) (Table 3). In both univariate and multivariate analysis, no prognostic factor was found for DMFS.

**Table 2:** Three-years survival rates of univariate analysis according to the prognostic factors

	LRFFS		DMFS		PFS		OS	
	%	*P*	%	*P*	%	*P*	%	*P*
FIGO stage		0.003		0.26		0.01		0.03
≤ IIA	100		100		100		100	
IIB	95.0		75.9		75.9		88.1	
III	71.3		65.0		46.0		52.6	
IV	50.0		100		50.0		100	
Node metastasis		0.42		0.15		0.14		0.09
Negative	95.2		83.3		79.2		91.7	
Positive	79.2		69.7		57.8		65.9	
Parametrial invasion		0.95		0.42		0.60		0.84
Positive	92.1		71.7		68.2		73.5	
Negative	75.0		90.9		68.1		88.9	
Tumor size		0.94		0.19		0.08		0.11
≤ 5 cm	90.0		80.6		71.7		79.2	
> 5 cm	84.1		68.0		61.0		56.8	
Initial tumor volume		0.39		0.76		0.32		0.53
< 65 cm^3^	86.5		75.7		67.3		62.4	
≥ 65 cm^3^	88.4		77.3		67.7		63.7	
HR-CTV		0.18		0.39		0.06		0.12
≤ 30 cm^3^	95.2		81.7		81.7		84.6	
> 30 cm^3^	75.9		68.8		49.6		70.0	
Chemothrapy		0.34		0.77		0.64		0.82
≤ 4 course	74.3		71.1		49.5		87.5	
≥ 5 course	94.4		78.2		76.2		73.9	
D90 (EQD2)		0.01		0.95		0.23		0.17
≤ 90 Gy	65.0		67.7		41.4		72.9	
> 90 Gy	100		78.6		78.6		80.2	

*LRFFS* loco-regional failure-free survival, *PFS* progression-free survival, *DMSF* distant metastasis-free survival, *OS* overall survival, *FIGO* Fédération Internationale de Gynécologie et d’Obstétrique, *HR-CTV* High risk clinical target volume

**Table 3:** Multivariate analysis for survivals

	LRFFS	DMFS	PFS	OS
FIGO Stage	0.04	0.09	0.01	0.02
Lymph node metastasis	0.29	0.63	0.56	0.67
High Risk CTV volume	0.19	0.70	0.57	0.52
D90 (EQD2)	0.93	0.85	0.60	0.60

*LRFFS* loco-regional failure-free survival, *PFS* progression-free survival, *DMFS* distant metastasis–free survival, *OS* overall survival

## Discussion

Radical RT in patients with cervix uteri carcinoma continues to improve with rapid developments in pre-treatment (staging) and treatment-related imaging technologies. Each patient should be accurately staged so that the appropriate treatment can be performed. Accurate staging improves local control and survival, while reducing morbidity and the failure associated with unnecessary radiation doses and inadequate irradiation, respectively [12]. Currently, FIGO staging serves as a clinical evaluation system. Clinical staging accuracy decreases with increasing stage (from 85 to 21%) [13]. Thus, where available, FIGO recommends cross-sectional imaging techniques. Although staging of cervix uteri carcinoma patients with CT is superior to clinical assessment, CT is less effective than both MRI and PET-CT [14, 15]. MRI is the best imaging technique for evaluating the invasion of primary tumor, showing high sensitivity (71–100%), specificity (88–91%), and negative predictive value (100%) [13]. However, PET-CT is superior to MRI for evaluating nodal involvement, showing high sensitivity (79–84% vs. 56–72%) and specificity (95–99% vs. 90–96%). In addition, PET-CT prompts changes in the RT field and treatment plan in 34 and 23% of patients, respectively. For these reasons, FIGO and the National Comprehensive Cancer Network (NCCN) recommend both MRI and PET-CT for pretreatment evaluation of cervix uteri carcinoma [12, 13]. In our study, all patients were imaged for staging and planning with both MRI and PET-CT before RT.

EBRT in patients with LACC is used to reduce the macroscopic tumor volume and eradicate subclinical disease, with acceptable toxicity. Currently, the optimal RT technique remains unknown. The 3D-CRT modality is commonly used and considered the gold standard in patients with LACC [16]. However, 3D-CRT pelvic four-field RT causes significant and acute toxicity in GIS and GUS, and in hematologic systems, which interrupts RT [17, 18]. Thus, the duration of RT is prolonged and the prognosis is negatively affected. For this reason, the clinical use of IMRT in patients with LACC has increased, especially during the last decade due to the known dosimetric advantages [19]. Currently, there are a limited number of clinical trials comparing the outcomes of IMRT and 3D-CRT usage in patients with LACC. Kidd et al. [20] and Yu et al. [21] reported significantly reduced acute grade 3 or higher treatment-related GI toxicity in their retrospective analyses of clinical results in patients with LACC treated with IMRT compared to the 3D-CRT technique (6% vs. 17%, *p* = 0.0017; and 5% vs. 30%, *p* < 0.05, respectively). However, unlike Yu et al. [21], Kidd et al. [20] reported a survival advantage with IMRT compared to the 3D-CRT technique. In addition, two randomized prospective studies, by Gandhi et al. [22] and Naik et al. [17], reported that IMRT reduced acute grade 3 or higher treatment-related GI toxicity (4.5% vs. 27.3%, *p* = 0.04; 5% vs. 20%, *p* = 0.004) without any survival advantage in patients with LACC. In our study, all of the patients were treated with 3D conformal EBRT, any grade 3 or higher treatment related toxicity was not observed both in GIS and GUS.

CHT is administered concurrently with RT to increase radiosensitivity. Although various concurrent CHT regimens have been used, the administration of DDP ± fluorouracil (FU) is now the recommended regimen, in accordance with the results of five randomized trials. In those trials, DDP was administered via the intravenous bolus route, weekly (40 mg/m^2^), triweekly (70–75 mg/m^2^), or every 4 weeks (50 mg/m^2^) [7–11]. Based on both randomized and retrospective trials, weekly DDP administration has been recommended due to similar survival rates versus other regimens but with fewer side-effects [23–25]. Although carboplatin (weekly; AUC 2) is recommended in patients who cannot be administered DDP, survival rates and tumor responses are lower than for DDP [26]. In our study, concurrent chemotherapeutic agent was DDP and administered 40 mg per square meter once a week to all patients, as recommended.

BRA is an integral part of radical RT in patients with LACC, and delivers high (> 80 Gy) radiation doses to the tumor, while sparing the OAR [27]. As with EBRT, there has been an improvement in image-guidance techniques, treatment planning technologies, and application systems over the last two decades. Image guidance techniques in BRA are two-dimensional (2-D) with plain-radiography, two and a half dimensional (2.5-D) with ultrasonography (USG) [28], and 3-D with CT and MRI. The 2-D BRA technique is considered the conventional technique, and is based on two points representing doses to the paracervical triangle (Point A) and pelvic wall (Point B), in accordance with the Manchester system. Worldwide, 2-D treatment has been the most commonly used modality over the last decade, because it can be applied in the BRA room and is inexpensive, easy to use, and does not require extensive planning time. However, tumor size and variations in adjacent OAR among patients are not considered. Thus, the treatment cannot be individualized. As a result, tumor control rates are decreased in large tumors and side-effects are increased in small tumors [28, 29]. In BRA, USG can be performed through both transabdominal and transrectal routes [30]. The advantages of both USG techniques are that they are economical, widely availability, portable, and have real-time applicability in the BRA room with relatively short application times. However, neither approach can assess target volume coverage, OAR, residual tumor, vaginal extension, or cumulative dose to the sigmoid colon. Therefore, if USG is used for planning, MRI should be performed to characterize the cervical tumor before RT. In addition, both USG techniques require operator experience and expertise [28, 31]. CT-based BRA planning is increasingly being implemented due to the use of CT-simulators for 3D-CRT in radiation oncology departments. Thus, delineation of the target volumes in consideration of at risk organs, and determining the accuracy of applicator placement, improves local control and survival without increasing acute and late morbidity. However, the most important disadvantage of CT is that it cannot adequately demonstrate the cervical tumor, including vaginal and parametrial extension [32]. Therefore, despite there being no difference between CT- and MRI-based delineation of at-risk organs, MRI is the gold standard 3D-imaging technique because it is optimal for soft tissue delineation for cervix uteri carcinoma, including primary tumor and soft tissue invasion. MRI is not routinely available in radiation oncology departments and requires specific applicators. CT-based BRA planning may involve only MRI fusion for planning, or post-EBRT pelvic MRI fusion for pre-planning [33, 34]. Second, BRA can be applied at an low dose rate (LDR) or HDR. Due to the disadvantages of LDR-BRA, such as radiation exposure to medical personnel, long treatment duration, and pulmonary embolism risk due to the long hospitalization, HDR-BRA has become widespread given that it has similar survival, recurrence and toxicity rates [35]. In addition, a wide range of applicators are used in BRA. The choice of the applicator depends on the anatomy of the individual patient and the tumor characteristics. The most commonly used applicators are tandem ovoid (TO) applicators with vaginal packing, and TR applicators with a rectal retractor. While there is no difference in tumor treatment outcomes between the two types of applicator, the radiation to which normal tissues are exposed is higher with the TO. The radiation dose to the rectum decreases by 12% using vaginal packing in TO. However, reports showed that the rectal retractor decreased the radiation dose to the rectum more so than did vaginal packing. Therefore, TR should be used with the rectal retractor if the patient anatomy and tumor characteristics are appropriate [36, 37]. In our study, 3-D conformal and HDR brachytherapy was applied with tandem and ring applicators to all patients.

Prognostic factors for survival include the FIGO stage, the presence of lymph node metastasis, D90 (EQD2) dose, and the HR-CTV. The 3-year OS and PFS were better for FIGO stages IB–IIB (OS = 83%, PFS = 87%) than IIIA–IVA (OS = 46%, PFS = 42%), and for lymph node-negative (OS = 77–92% and PFS = 85%) versus -positive status (OS = 50–72%, PFS = 53%) [38, 39]. Improved 3-year local control rates were reported with ≥87 Gy doses of D90-HR-CTV (96% vs. 80% for D90-HR-CTV < 87 Gy) and with ≤30 cm^3^ or smaller HR-CTV volumes (92% vs. 72% for > 30 cm^3^ volumes) [40, 41]. In our study, the most important prognostic factor was FIGO stage and 3-year LRFFS rate was found better with > 90 Gy doses of D90-HR-CTV (100% vs. 65% for D90-HR-CTV ≤ 90 Gy; *P* = 0.01).

Comparing image-guided BRA (IGA-BRA) and conventional BRA, survival improved with the former (3-year OS = 86% vs. 51%, respectively) and grade 3 or higher late toxicity decreased (8% vs. 15%, respectively) [42]. According to the IGA-BRA trials, the 3-year PFS, OS, and late toxicity rates are 65–86%, 74–87%, and 5–9.5%, respectively. Lastly, the majority of deaths in patients with cervix uteri cancer after radical RT are due to distant metastases [38, 39, 42–44]. In our study with using three dimensional conformal BRA; the 3-year overall survival was 76.9% and distant metastasis was the most common cause of death. These findings were consistent with the literature.

In the present study, we reported image-guided radical RT results in patients with LACC drawn from the middle Black Sea region of Turkey. To the best of our knowledge, this is the first Turkish study using both EBRT and intracavitary BRA image-guided RT techniques. We found that the most important prognostic factor was the FIGO stage. Our PFS and OS results are consistent with previous reports. Although the rate of late toxicity has been reported to be between 5 and 9.5%, we did not detect any late toxicity in our patients. In addition, as in previous reports, distant metastasis was the most common cause of death in patients with LACC despite radical RT.

Limitations of the present study included its retrospective nature, single institution design, small number of patients, and short follow-up time.

## Conclusions

Cervix uteri carcinoma was diagnosed in the advanced stage in our patients from the Black Sea region of Turkey, typical of developing countries. However, the treatment and survival outcomes were as good as those in developed countries because treatment involved both image-guided 3-D EBRT and intracavitary BRA. Recurrence was mostly in the form of distant metastases and further investigations on systemic therapies are required.

## Data Availability

The datasets used and analyzed during the current study are available from the corresponding author on reasonable request.

## Abbreviations

3D: Three dimensional
BRA: Brachytherapy
CHT: Chemotherapy
CrCl: Creatinine clearance
CRT: Conformal radiotherapy
CT: Computed tomography
CTV: Clinical target volume
DDP: Cisplatin
DMFS: Distant metastasis-free survival
EBRT: External beam radiotherapy
EORTC: European Organization for Research and Treatment of Cancer
FIGO: Fédération Internationale de Gynécologie et d’Obstétrique
GEC-ESTRO: Groupe Européen de Curiethérapie and the European Society for Radiotherapy and Oncology
GIS: Gastrointestinal system
GTV: Gross tumor volume
GUS: Genitourinary system
HDR: High dose rate
HR-CTV: High-risk clinical target volume
IGA: Image guided
LACC: Locally advanced cervix uteri carcinoma
LDR: Low dose rate
LRFFS: Locoregional failure-free survival
MRI: Magnetic resonance imaging
NCCN: National Comprehensive Cancer Network
OAR: Organs at risk
OS: Overall survival
PET-CT: Positron emission tomography-computed tomography
PFS: Progression-free survival
PTV: Planning target volume
RT: Radiotherapy
RTOG: Radiation Therapy Oncology Group
TO: Tandem ovoid
TR: Tandem ring
USG: Ultrasonography
